# Oxaliplatin-Induced Lhermitte's Sign in Gastric Cancer

**DOI:** 10.1155/2020/8826657

**Published:** 2020-06-24

**Authors:** Takeshi Okamoto, Koichi Takagi, Katsuyuki Fukuda

**Affiliations:** Department of Gastroenterology, St. Luke's International Hospital, 9-1 Akashicho, Chuo-ku, Tokyo 104-8560, Japan

## Abstract

A 64-year-old Japanese man with no significant medical history presented with epigastric discomfort of 2-weeks' duration. He was diagnosed with metastatic HER2-positive gastric cancer. Chemotherapy with capecitabine, oxaliplatin, and trastuzumab was initiated. During the eighth cycle, he suddenly complained of electric shock-like pain in both legs upon neck flexion, consistent with Lhermitte's sign. Oxaliplatin was discontinued, and Lhermitte's sign resolved after 3 months. Neurotoxicity is commonly observed in platinum-based chemotherapy, but Lhermitte's sign is rare. This is the first report of oxaliplatin-induced Lhermitte's sign in a gastric cancer patient.

## 1. Introduction

Lhermitte's sign is an electric shock-like sensation of the upper and/or lower extremities triggered by flexion of the neck. It was first described by Marie and Chatelin during World War I and reported by Lhermitte et al. in 1924 [1, 2]. While most commonly observed in multiple sclerosis, it has also been reported in several conditions including spinal cord tumors, herpes zoster, systemic lupus erythematosus, Behcet's disease, and vitamin B12 deficiency [3–7]. Lhermitte's sign is also associated with radiotherapy and chemotherapy, with cisplatin being the most commonly reported causative agent and several reports of other agents including oxaliplatin, carboplatin, docetaxel, busulfan, and cyclophosphamide [8–14]. We report the first case of oxaliplatin-induced Lhermitte's sign in a gastric cancer patient.

## 2. Case Presentation

A 64-year-old Japanese man with no significant medical history presented with epigastric discomfort of 2-weeks' duration. Esophagogastroduodenoscopy revealed a 2 cm mass at the gastroesophageal junction. He was diagnosed with HER2-positive gastric cancer with lymph node and lung metastases. Triweekly chemotherapy with capecitabine (2000 mg/m^2^ for 14 days followed by 7 days of rest), oxaliplatin (130 mg/m^2^ every 21 days) and trastuzumab (initial dose of 8 mg/kg and 6 mg/kg every 21 days thereafter) was initiated.

The patient began to complain of mild transient numbness and tingling in his fingers and toes during the third cycle. The peripheral neuropathy remained mild (Grades 1-2 neurotoxicity) despite continued therapy at the full dose up to the eighth cycle. After the eighth cycle, he suddenly complained of electric shock-like pain in both legs upon neck flexion. The pain occurred instantaneously after neck flexion, but not by neck rotation. The patient complained that he could not sleep because using a pillow would induce the pain. The pain was strongest in his thighs and involved the entire lower extremities but spared the upper extremities. He had difficulty turning the pages of a book and buttoning his shirt. He could still turn doorknobs and use chopsticks but would drop them from time to time. He also noted taste impairment. He denied any recent head and neck trauma or urinary or fecal incontinence. On physical examination, the patient was in his normal state of health and consciousness. No ulcers, sores, rashes, or other findings suggestive of herpes zoster, systemic lupus erythematosus, or Behcet's disease were noted. Cranial and peripheral nerve examinations were only significant for decreased deep tendon reflexes. No abnormal reflexes or sensory disturbance below a certain level suggesting cervical spinal cord injury was observed. The cumulative oxaliplatin dose was 1040 mg/m^2^.

**Figure 1:**
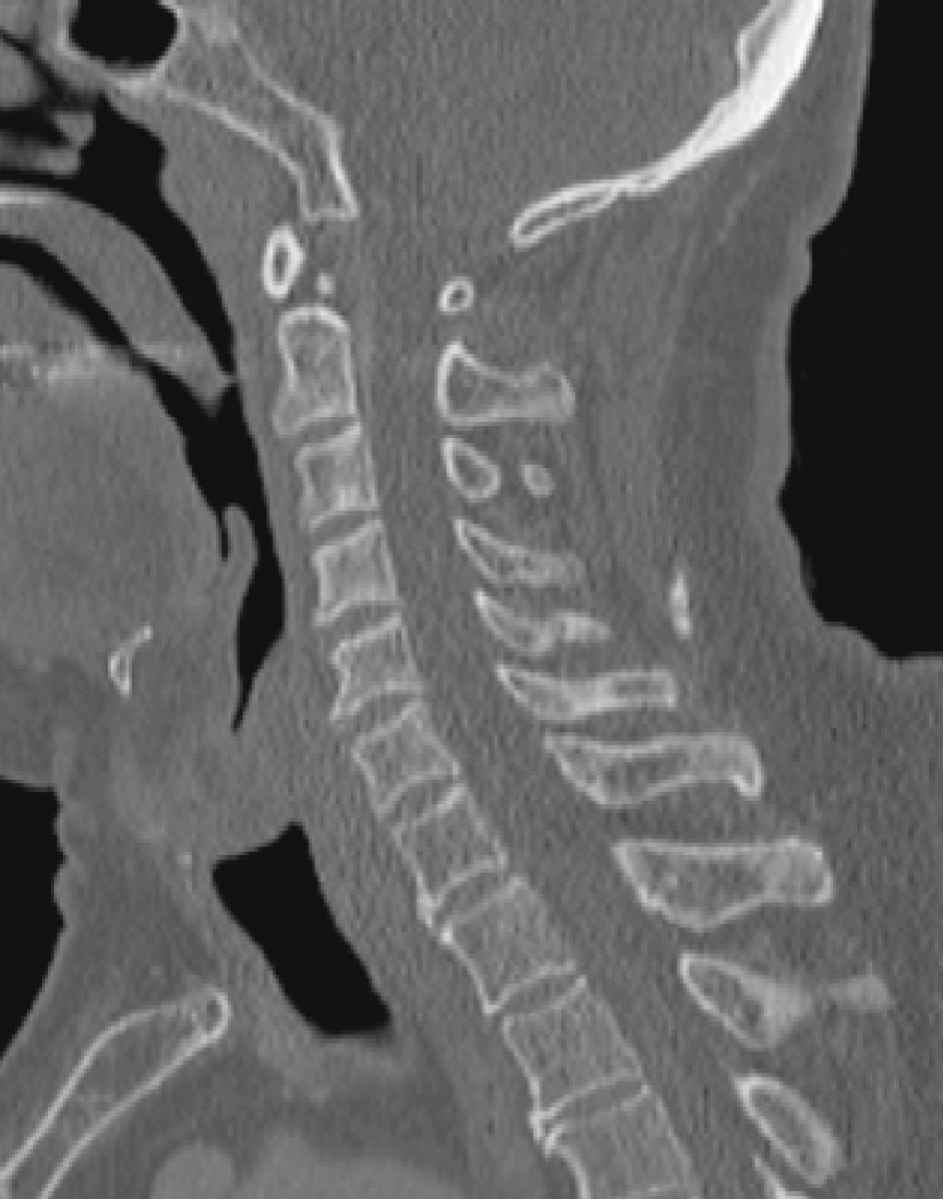
CT of the cervical spine showed no abnormalities.

Laboratory data was only significant for an increase in carcinoembryonic antigen from 9.5 to 16.3 ng/mL. White blood cells and C-reactive protein were normal, making infection an unlikely etiology. While the patient was a heavy drinker, magnesium, vitamin B12, and nutritional markers such as albumin were all within the normal range. Computed tomography (CT) showed slight enlargement of lung metastases and small low density areas in the liver suggesting possible metastases. However, no bone metastases or displacement were noted in the cervical spine or elsewhere and no masses in the spinal column were found (Figure 1). The patient was not taking any oral medications at the time. The patient was diagnosed with oxaliplatin-induced Lhermitte's sign (OLS).

**Figure 2:**
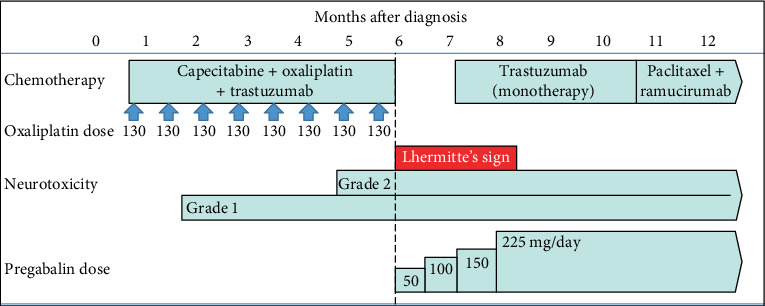
Course of treatment during the first year after diagnosis of gastric cancer.

Pregabalin was started at 50 mg/day and increased gradually to 225 mg/day. Some improvement of Lhermitte's sign was observed after a one-month drug holiday requested by the patient. OLS resolved completely within 3 months after termination of oxaliplatin, while the patient was on trastuzumab monotherapy (Figure 2). Subsequent chemotherapy included paclitaxel, ramucirumab, nivolumab, and trifluridine/tipiracil. Grade 2 neurotoxicity persisted until his death 20 months later.

## 3. Discussion

Lhermitte's sign is a rare condition which has been described to result from stretching the hyperexcitable dorsal column of the cervical spinal cord after demyelination [15]. It can be triggered by neck movements, fatigue, stress, and heat [2]. While Lhermitte's sign is diagnosed on clinical grounds, laboratory and imaging studies are required to determine its cause.

Oxaliplatin is known to cause 2 types of peripheral neuropathy [16]. The first is an acute, temporary paresthesia which occurs during or soon after infusion, characteristically induced by exposure to cold temperature. The second is a cumulative, dose-dependent sensory peripheral neuropathy. This dose-limiting toxicity occurs in 16% of patients exposed to at least 765 mg/m^2^ of oxaliplatin, which reverses within a few months after cessation in a majority of patients [16, 17]. The risk of neuropathy increases in patients with diabetes mellitus, chronic alcoholism, or the use of other potentially neurotoxic agents [10]. OLS has been reported to occur only in the presence of cumulative sensory neuropathy [12, 18–21].

Magnetic resonance imaging (MRI) of the cervical spine is indicated to rule out spinal cord injury. Demyelination is more likely to be detected in multiple sclerosis patients with Lhermitte's sign than those without [22]. On the other hand, no MRI abnormalities were noted in a study of 5 OLS patients [12]. Nerve conduction studies can show generalized axonal-type sensory neuropathy, especially in the sural nerve amplitude [12]. MRI was not performed for our patient as he refused to visit the hospital during his drug holiday. However, the lack of dermatomal neurological findings and recovery after discontinuation of oxaliplatin are inconsistent with spinal cord injury. The low initial dose of pregabalin may have provided limited relief but had no favorable effect on his peripheral neuropathy and would be extremely unlikely to reverse Lhermitte's sign if spinal cord injury had been the cause.

While our treatment regimen also included capecitabine and trastuzumab, peripheral neuropathy from both drugs is very rare [23, 24]. Reported neurological symptoms from capecitabine include pain, tingling, or numbness mainly in the legs, with no reports of Lhermitte's sign [23]. There were no cases of Grade 3 or 4 neurotoxicity reported in a randomized control trial in which 1694 patients received trastuzumab monotherapy [25]. Lhermitte's sign resolved in our patient despite continuing trastuzumab, ruling out trastuzumab-induced peripheral neuropathy. We therefore determined that oxaliplatin was the causative agent.

OLS is extremely rare, possibly due to underdiagnosis and underreporting. A total of 14 cases have been reported in the English literature, with 13 cases of colorectal cancer summarized in a literature review by Amaraneni et al. and the other being an ovarian cancer patient treated with cisplatin before using oxaliplatin [12, 18–21]. OLS observed in the 13 colorectal cancer patients all resulted from 5-fluorouracil-based regimens including oxaliplatin. Recovery from OLS was seen in 11 of 13 cases in 2-17 months, while the other 2 had unresolved OLS at death (3 and 6 months, respectively). The cumulative dose (1040 mg/m^2^) and time to recovery after discontinuation of oxaliplatin (3 months) in our case was comparable to the colorectal cancer cases (Table 1). On the other hand, the peripheral neuropathy observed in this patient when Lhermitte's sign was first diagnosed appeared milder than past reports.

Despite 13 reports of OLS arising in colorectal cancer, this is the first report of OLS in a gastric cancer patient. It is unclear whether OLS is less likely to occur in chemotherapy for gastric cancer than for colorectal cancer. Several explanations may be offered. First, gastric cancer is rarer than colorectal cancer in North America and Europe. For example, the age-standardized incidence rate of colorectal cancer in North America is about 6 times higher than that of gastric cancer [26]. On the other hand, no reports of OLS in gastric cancer patients were found in a search of the Japanese literature (on Ichushi) despite a similar number of gastric and colorectal cancer patients in Japan. Second, the faster progression and worse prognosis of gastric cancer may make it less likely for patients to reach the dose-limiting toxicity before experiencing progressive disease [27]. Third, XELOX (capecitabine+oxaliplatin) is used more commonly in gastric cancer than FOLFOX (5-fluorouracil+folinic acid+oxaliplatin), which is frequently used in colon cancer. Hand-foot syndrome occurs more commonly which results from capecitabine than from intravenous 5-fluorouracil [28]. As both hand-foot syndrome and peripheral neuropathy affect the distal extremities, patients may be more likely to complain of adverse effects from regimens including both capecitabine and oxaliplatin, leading physicians to reduce or discontinue oxaliplatin despite relatively mild neuropathy. While a direct comparison must be viewed with caution, a Japanese study on adjuvant FOLFOX for colorectal cancer [29] reported median oxaliplatin dose intensity of 79.5% over 24 weeks, while a phase II Japanese study on adjuvant XELOX for gastric cancer [30] reported mean oxaliplatin dose intensity of 73.4% (95% CI, 68.4-78.4%). Fourth, gastric cancer patients on XELOX generally receive 130 mg/m^2^ of oxaliplatin every 21 days, while colorectal cancer patients on FOLFOX receive 85 mg/m^2^ every 14 days. While the cumulative dose over the same time period is similar (1,040 mg/m^2^ with XELOX and 1,020 mg/m^2^ with FOLFOX after 24 weeks), more frequent outpatient visits may create more opportunity for patients to complain of adverse effects and for physicians to notice them and reduce or discontinue oxaliplatin accordingly. Furthermore, the lack of neutropenia or other adverse effects in our patient led him to receive the full dose for 8 cycles, increasing the likelihood of oxaliplatin-induced neuropathy. Data from more patients are required to determine whether OLS is more likely to occur in colorectal cancer patients than in gastric cancer patients.

OLS is generally self-limited, and treatment mainly involves discontinuing oxaliplatin. Pharmaceutical treatment options include carbamazepine, gabapentin, pregabalin, and duloxetine, although evidence is scarce [31–34]. It is unclear whether stop-and-go strategies for oxaliplatin can reduce the likelihood of OLS occurrence, as no OLS was reported and as no significant reduction in severe sensory neuropathy was found in a randomized study [35].

Lhermitte's sign should be interpreted as a dose-limiting neurotoxicity. While symptoms associated with Lhermitte's sign are severe and patients will generally complain of them as soon as they appear, oncologists must remain aware of this possibility when using platinum-based regimens.

## Figures and Tables

**Table 1:** Comparison between our case and 13 colorectal cancer patients with OLS.

	Gastric cancer	Colon cancer	
Number of reported cases	1 (our case)	13	
Chemotherapy regimens	XELOX+trastuzumab	FOLFOX (*n* = 12) XELOX (*n* = 1)	
Age	64	54.5 (28-73)	(average, range)
Gender	Male	46.2% male	
Cumulative oxaliplatin dose (mg/m)	1040	1061 (574-2040)	(average, range)
Time to recovery (months)	3	6.5^∗^ (2-17)	(average, range)

^∗^Average of 10 cases with precise reports of time to recovery (in months).

## Data Availability

Data available upon request, contact Takeshi Okamoto at okamotot@luke.ac.jp.
